# Prepubertal exposure to zearalenone or genistein reduces mammary tumorigenesis

**DOI:** 10.1038/sj.bjc.6690584

**Published:** 1999-08

**Authors:** L Hilakivi-Clarke, I Onojafe, M Raygada, E Cho, T Skaar, I Russo, R Clarke

**Affiliations:** Lombardi Cancer Center, Department of Psychiatry and Department of Physiology, Georgetown University, Washington, DC 20007, USA; Breast Cancer Research Laboratories, Fox Chase Cancer Center, Philadelphia, PA 19111, USA

**Keywords:** genistein, zearalenone, prepuberty, mammary tumorigenesis

## Abstract

Prepubertal exposure to a pharmacological dose (500 mg kg^−1^) of the phyto-oestrogen genistein can reduce the incidence and multiplicity of carcinogen-induced mammary tumours in rats. However, such an exposure also disrupts the function of the hypothalamic–pituitary–gonadal axis, making it unsuitable for breast cancer prevention. We studied whether prepubertal exposure to genistein at a total body dose broadly comparable to the level typical of Oriental countries, approximately 1 mg kg^−1^ body weight, affects mammary tumorigenesis. We also studied whether prepubertal exposure to zearalenone, a major source for phyto-oestrogens in the USA, influences breast cancer risk. Prepubertal rats were treated between postnatal days 7 and 20, with 20 μg (~ 1 mg kg^−1^ body weight) of either genistein or zearalenone. Zearalenone exposure significantly reduced both the incidence and multiplicity of mammary tumours induced by 7,12-dimethylbenz(a)anthracene (DMBA). Genistein exposure significantly reduced tumour multiplicity, but not tumour incidence, when compared with vehicle-treated animals. Furthermore, 60% of the tumours in the genistein group were not malignant, while all the tumours analysed for histopathology in the vehicle and zearalenone groups were adenocarcinomas. A higher number of differentiated alveolar buds, and lower number of terminal ducts, were present in the DMBA-treated mammary glands of the phyto-oestrogen exposed rats. The concentration of oestrogen receptor (ER) binding sites after the DMBA treatment was low in the mammary glands of all groups but a significantly higher proportion of the glands in the zearalenone exposed rats were ER-positive (i.e. ER levels ≥ 5 fmol mg^−1^ protein) than the glands of the vehicle controls. Our data suggest that a prepubertal exposure to a low dose of either zearalenone or genistein may protect the mammary gland from carcinogen-induced malignant transformation, possibly by increasing differentiation of the mammary epithelial tree. © 1999 Cancer Research Campaign


*British Journal of Cancer* (1999) **80**(11), 1682–1688
© 1999 Cancer Research Campaign
Article no. bjoc.1999.0584

### Prepubertal exposure to zearalenone or genistein reduces mammary tumorigenesis

**L Hilakivi-Clarke[1,2], I Onojafe[1], M Raygada[1,2], E Cho[1,3], T Skaar[1,3], I Russo[4] and R Clarke[1,3]**

[1]Lombardi Cancer Center, [2]Department of Psychiatry and [3]Department of Physiology, Georgetown University, 3970 Reservoir Rd NW, Washington, DC 20007, USA; [4]Breast Cancer Research Laboratories, Fox Chase Cancer Center, Philadelphia, PA 19111, USA

**Summary** Prepubertal exposure to a pharmacological dose (500 mg kg$^{-1}$) of the phyto-oestrogen genistein can reduce the incidence and multiplicity of carcinogen-induced mammary tumours in rats. However, such an exposure also disrupts the function of the hypothalamic–pituitary–gonadal axis, making it unsuitable for breast cancer prevention. We studied whether prepubertal exposure to genistein at a total body dose broadly comparable to the level typical of Oriental countries, approximately 1 mg kg$^{-1}$ body weight, affects mammary tumorigenesis. We also studied whether prepubertal exposure to zearalenone, a major source for phyto-oestrogens in the USA, influences breast cancer risk. Prepubertal rats were treated between postnatal days 7 and 20, with 20 μg (~ 1 mg kg$^{-1}$ body weight) of either genistein or zearalenone. Zearalenone exposure significantly reduced both the incidence and multiplicity of mammary tumours induced by 7,12-dimethylbenz(a)anthracene (DMBA). Genistein exposure significantly reduced tumour multiplicity, but not tumour incidence, when compared with vehicle-treated animals. Furthermore, 60% of the tumours in the genistein group were not malignant, while all the tumours analysed for histopathology in the vehicle and zearalenone groups were adenocarcinomas. A higher number of differentiated alveolar buds, and lower number of terminal ducts, were present in the DMBA-treated mammary glands of the phyto-oestrogen exposed rats. The concentration of oestrogen receptor (ER) binding sites after the DMBA treatment was low in the mammary glands of all groups but a significantly higher proportion of the glands in the zearalenone exposed rats were ER-positive (i.e. ER levels ≥ 5 fmol mg$^{-1}$ protein) than the glands of the vehicle controls. Our data suggest that a prepubertal exposure to a low dose of either zearalenone or genistein may protect the mammary gland from carcinogen-induced malignant transformation, possibly by increasing differentiation of the mammary epithelial tree.

**Keywords:** genistein; zearalenone; prepuberty; mammary tumorigenesis

Asian populations with a high intake of phyto-oestrogens have a relatively low incidence of breast cancer (Setchell et al, 1984). Therefore, it has been suggested that phyto-oestrogens may reduce breast cancer risk (Messina et al, 1994). Phyto-oestrogens are naturally occurring compounds produced by a variety of plants. They are present in several foods, including soybean-based products that are typical for diets of Asia, but not for Western countries. Approximately 50% of the isoflavones in soybeans consists of genistein (Messina et al, 1994). There is some epidemiological evidence in favour of soy/genistein being anti-tumorigenic, but this is controversial. In three of a total of eight studies, a statistically significant association between high soy intake and low breast cancer risk has been found (Nomura et al, 1978; Hirohata et al, 1985; Lee et al, 1991; Yuan et al, 1995; Wu et al, 1996; Witte et al, 1997; Zheng et al, 1999). While most animal studies are supportive of the hypothesis that genistein inhibits mammary tumour promotion (Hawrylewicz et al, 1991; Messina et al, 1994; Barnes, 1997; Gotoh et al, 1998), some studies show an ability of genistein to increase mammary tumorigenesis (Hsieh et al, 1998). Prepubertal exposure via injections to a pharmacological dose of genistein (500 mg kg$^{-1}$) on days 16, 18 and 20 is reported to dramatically reduce subsequent risk to develop mammary tumours (Murrill et al, 1996). Thus, genistein may be particularly effective in reducing breast cancer risk, if its exposure occurs prior to puberty.

Zearalenone is another phyto-oestrogen that may be linked to mammary tumorigenesis. Zearalenone is mainly produced by the mould *Fusarium graminearum* found in a variety of host plants and debris from soil around the world (Burgess et al, 1982). It is present as a contaminant in stored cereals, being found in barley, corn, corn flakes, rice and wheat at concentrations from 35 to 115 μg kg$^{-1}$ (Hagler et al, 1984; Schoental, 1985; Luo et al, 1990). Zearalenone also is used as an anabolic agent to enhance growth in cattle and lambs (Ralston, 1978; Wiggins et al, 1979). In contrast to genistein, pharmacological doses (10 mg kg$^{-1}$ body weight) of zearalenone have been associated with an increased breast cancer risk in rats (Schoental, 1974).

Enthusiasm concerning the effects of prepubertal genistein exposure is limited because the dose used in previous studies is 5000 times higher than that of human exposure on a mg kg$^{-1}$ body weight basis (Murrill et al, 1996). This pharmacological exposure causes severe perturbations in hypothalamic-ovarian function (Murrill et al, 1996) that may lead to infertility. Asians consume 2–9 g soy protein daily, while most women in Western countries do not consume any soy (Seow et al, 1998; Wu et al, 1998).

*Received 6 April 1998*
*Revised 16 February 1999*
*Accepted 17 February 1999*

*Correspondence to:* L Hilakivi-Clarke

This work was supported by grants from the American Cancer Society (CN-80420), and the Lombardi Cancer Center Shared Animal Resource Facility, U.S. Public Health Service Grant 2P30-CA51008.

Genistein content of different soy products varies considerably; for example, soy beans, soy milk and tofu contain approximately 2–18 μg genistein per g product, while miso and natto contain 40–330 μg genistein per g product (Lu et al, 1995; Fukutake et al, 1996). Fukutake et al (1996) have estimated that the daily genistein intake is 1.5–4.1 mg (< 0.1 mg $kg^{-1}$) among Asians. Zearalenone is the main phyto-oestrogen consumed in the USA (Kuiper-Goodman, 1990). The present study examined whether a prepubertal exposure to a more physiological dose of genistein and zearalenone (~ 1 mg $kg^{-1}$ body weight) alters carcinogen-induced mammary tumorigenesis in rats. In addition, we investigated whether the concentrations of total oestrogen receptor (ER) binding sites are altered by prepubertal phyto-oestrogen administration. Low doses of genistein and zearalenone are known to bind to ER and affect its transcriptional regulatory activities (Wang et al, 1996; Collins et al, 1997; Zava and Duwe, 1997). The effect on mammary gland morphology also was studied, since a previous report suggested that an increased differentiation of the mammary epithelial tree by prepubertal genistein exposure may explain its cancer-reducing effects (Murrill et al, 1996).

### METHODS

### Animals

Pregnant female Sprague-Dawley rats, purchased from Charles Rivers Breeding Laboratories, were obtained at day 10 of gestation. The animals were housed singly, in standard rat plexiglas cages, at a constant temperature (20–22°C) and humidity (60–65%), under a 12-h light–dark cycle (lights on 06:00 h). Two days after the offspring were born, the males were sacrificed and the females cross-fostered. Ten to twelve female pups were housed with a lactating dam. The female offspring were weaned on postnatal day 22, and thereafter housed in groups of 3–5 animals. All studies were performed in accordance with the appropriate institutional and federal requirements.

### Phyto-oestrogen exposure

On postnatal day 7, the litters were divided into three groups. The offspring received 20 μg genistein, 20 μg zearalenone (both from Sigma Chemical Co., St Louis, MO, USA), or vehicle, administered as subcutaneous injections, in a volume of 0.05 ml. Phyto-oestrogens were first dissolved in 2% dimethyl sulphoxide (DMSO), and then mixed with peanut oil (this mixture also served as vehicle). The injections were repeated on days 10, 14, 17 and 20. Body weight of a rat pup on day 7 is approximately 10 g and on day 20 25–30 g. Thus, the animals received genistein at the doses ranging from 2 mg $kg^{-1}$ (day 7) to 0.7 mg $kg^{-1}$ (day 20).

### Inducing and monitoring of mammary tumorigenesis

Mammary tumors were induced by administration of 10 mg (~ 50 mg $kg^{-1}$ body weight) 7,12-dimethylbenz(a)anthracene (DMBA) (Sigma, St Louis, MO, USA). This is a suboptimal dose used in our laboratory to enable assessments of both reductions and increases in the end points of tumorigenicity (Hilakivi-Clarke et al, 1997*b*). More than 75% of the tumours induced by 10 mg DMBA are adenocarcinomas (Russo and Russo, 1987). The carcinogen was dissolved in peanut oil and administered by oral gavage in a volume of 1 ml. Animals were 45 days old at the time of DMBA administration. The groups that were given phyto-oestrogens or vehicle after birth each contained 30 animals.

The animals were examined regularly for mammary tumours by palpation once per week. The end points for data analysis were (i) latency to tumour appearance, (ii) the number of animals with tumours (tumour incidence), (iii) the number of tumours per animal (tumour multiplicity), and (iv) tumour proliferation. A tumour was designated as proliferating if it increased regularly in size. Tumour sizes were measured by recording the tumour diameters with a caliper and determining the length of the longest axis and the width perpendicular to the longest axis. The animals were sacrificed when detectable tumour burden approximated 10% of total body weight, as required by our institution. All surviving animals, including those that did not appear to develop mammary tumours, were sacrificed 19 weeks after carcinogen administration.

Histopathology of the DMBA-induced mammary tumours was evaluated from 22 haematoxylin and eosin stained samples. Two pathologists at the Lombardi Cancer Center (Georgetown University) independently assessed the tumour samples. The pathologists were blind to the experimental groups.

### Oestrogen receptor

The number of ER binding sites in the 4th mammary glands were determined from female rats exposed to genistein, zearalenone, or vehicle during the prepubertal period ($n$ = 7 per group). The animals from which the mammary glands were taken had been treated with DMBA 18 weeks before, and consequently developed at least one mammary tumour. None of the tumours were in the 4th gland in the animals used for ER assays. ERs were detected using a ligand binding assay as described by Nelson et al (1986). The ligand used was [2,4,6,7-$^3$H] 17β oestradiol (specific activity 99 Ci $mmol^{-1}$; Amersham, Arlington Heights, IL, USA). This assay detects both ERα and ERβ with equal efficacy. Thus, ER binding reflects total ER concentrations (ERα + ERβ).

### Mammary gland morphology

Whole mounts of the 9th mammary glands of the same female rats whose 4th glands were used for ER assays, were prepared ($n$ = 4–5 per group). At the time of sacrifice, 18 weeks had passed from the DMBA administration. The removed glands were stained with carmine aluminium as previously described by Haslam (1988). We have previously validated a visual scale to study the development of a mouse and rat mammary epithelial tree (Hilakivi-Clarke et al, 1997*a*, 1997*b*). Using this scale, we determined differentiation of mammary epithelial structures in the whole mounts. The mammary epithelial trees were analysed for the density of ductal structures, terminal ducts and differentiated alveolar buds. This analysis was done double-blind under an Olympus dissecting microscope, using a 5-point scale (from 0 = absent to 5 = numerous). Differentiated alveolar buds do not give rise to adenocarcinomas. While terminal ducts occasionally give rise to tumours, the majority of tumours originate from terminal end buds (Russo and Russo, 1987). However, at the time the whole mounts were obtained from 6-month-old rats, all terminal end buds had differentiated to alveolar buds or regressed to terminal ducts. In addition to this quantitative analysis of the mammary whole mounts, a qualitative evaluation was performed.

*© 1999 Cancer Research Campaign*

**Table 1** Effects of early postnatal exposure to 20 µg genistein or 20 µg zearalenone on mammary tumour growth

| | Tumour latency (weeks) | Tumour area (mm²) | Tumour multiplicity | Number of non-proliferating tumours |
|---|---|---|---|---|
| Vehicle (*n* = 33/17)[d] | 11.0 ± 0.7 | 64.5 ± 13.5 | 1.8 ± 0.3 | 2 (6%) |
| Genistein (*n* = 15/13) | 11.4 ± 0.3 | 67.7 ± 14.2 | 1.1 ± 0.1[b] | 6 (40%)[c] |
| Zearalenone (*n* = 13/9) | 14.4 ± 0.3[b] | 58.7 ± 15.7 | 1.2 ± 0.1[a] | 5 (38%)[c] |

Significantly different from vehicle group: [a]$P < 0.06$, [b]$P < 0.01$, [c]$P < 0.001$. [d]*n* = Number of tumours/number of animals with tumours. Note: 60% of the tumours in the genistein group are benign, while all tumours examined in the vehicle and zearalenone groups are malignant adenocarcinomas. Data represent the mean ± s.e.m. of latency to tumour appearance, area of tumours at first detection, tumour multiplicity and percentage of non-proliferating tumours. Number of rats per group = 30.

### Statistical analyses

Results for the data obtained on weight gain, ER binding sites, density of epithelial ducts, terminal ducts and alveolar buds in the whole mounts, and tumour latency, multiplicity, size upon first detection and growth data were analysed using one-way analysis of variance (Snedecor, 1988; Hanfelt, 1997). Where appropriate, between-group comparisons were done using Fisher's least significant difference. Results of tumour incidence were analysed using a log-rank survival analysis test. Differences in the number of non-proliferating and proliferating tumours, and the percentage of mammary glands that contained ER levels that were either ≥ 5 fmol mg$^{-1}$ protein or < 5 fmol mg$^{-1}$ protein, were determined using a $\chi^2$ test. All probabilities are two-tailed. Statistical tests were performed using the BMDP software (BMDP Statistical Software, Los Angeles, CA, USA).

### RESULTS

### Effect on weight gain

Early postnatal exposure to either genistein or zearalenone did not affect body weight gain. Between the first (day 7) and last day of phyto-oestrogen exposure (day 21), weight increased by 2.5-fold in the vehicle-treated rats, by 2.5-fold in the genistein-treated rats and by 2.6-fold in the zearalenone-treated rats. Body weights also were similar at the time the carcinogen was administered or 18 weeks after the administration (data not shown).

### Mammary tumorigenesis

### *Tumour latency*

The first tumours appeared on week 7 after the DMBA exposure in all groups. The mean tumour latency time to the first tumour per animal was significantly longer in the female rats exposed to zearalenone during early postnatal period than in the vehicle-treated rats ($F(2,37) = 4.91$, $P < 0.01$) (Table 1). Tumour latency was similar in the rats exposed to genistein or vehicle during prepuberty.

### *Tumour incidence*

The incidence of mammary tumours (number of animals with tumours per group) was determined weekly, beginning on week 7 following DMBA administration. At the end of the study, on week 18 following DMBA administration, the percentage of rats with mammary tumours was 57% (17/30) in the vehicle-treated group, 43% (13/30) in the genistein-treated group, and 30% (9/30) in the zearalenone-treated group ($\chi^2 = 14.92$, df = 2, $P < 0.001$).

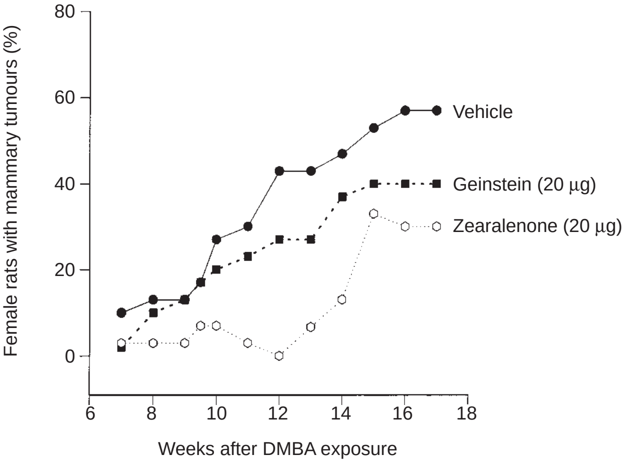

**Figure 1** The proportion of female rats exposed to 20 µg genistein, 20 µg zearalenone, or vehicle during postnatal days 7, 10, 14, 17 and 20, that developed DMBA-induced (administered on day 45) mammary tumours. The number of animals per group was 30. Tumour incidence was significantly lower in the zearalenone-treated rats ($P < 0.001$)

Log-rank test analysis indicated that the tumour incidence was significantly lower in the animals exposed to zearalenone during the prepubertal period than in the vehicle-treated controls (z-value = 2.36, $P < 0.018$). The slightly lower tumour incidence in the genistein-exposed rats was not statistically significant, when compared with the controls (z = 1.03, $P < 0.30$) (Figure 1).

### *Tumour multiplicity*

The average number of tumours per animal was significantly lower in the rats exposed to genistein ($P < 0.01$) during prepubertal life than in the vehicle-treated rats ($F(2,38) = 4.53$, $P < 0.02$). A reduction that approached statistical significance also was seen in the zearalenone-exposed rats ($P < 0.06$) (Table 1).

### *Tumour growth rate*

The size of the tumours upon first detection was similar among the groups (Table 1). However, the percentage of proliferating tumours in the genistein- (60%) and zearalenone-exposed rats (62%) was significantly lower than in the vehicle-treated group (94%) ($\chi^2 = 9.96$, df = 2, $P < 0.001$).

### *Tumour histopathology*

Histopathological analysis performed for 22 samples indicated that the histotypes of all mammary tumours in the vehicle- and zearalenone-treated groups were adenocarcinomas (100%). In the

© 1999 Cancer Research Campaign

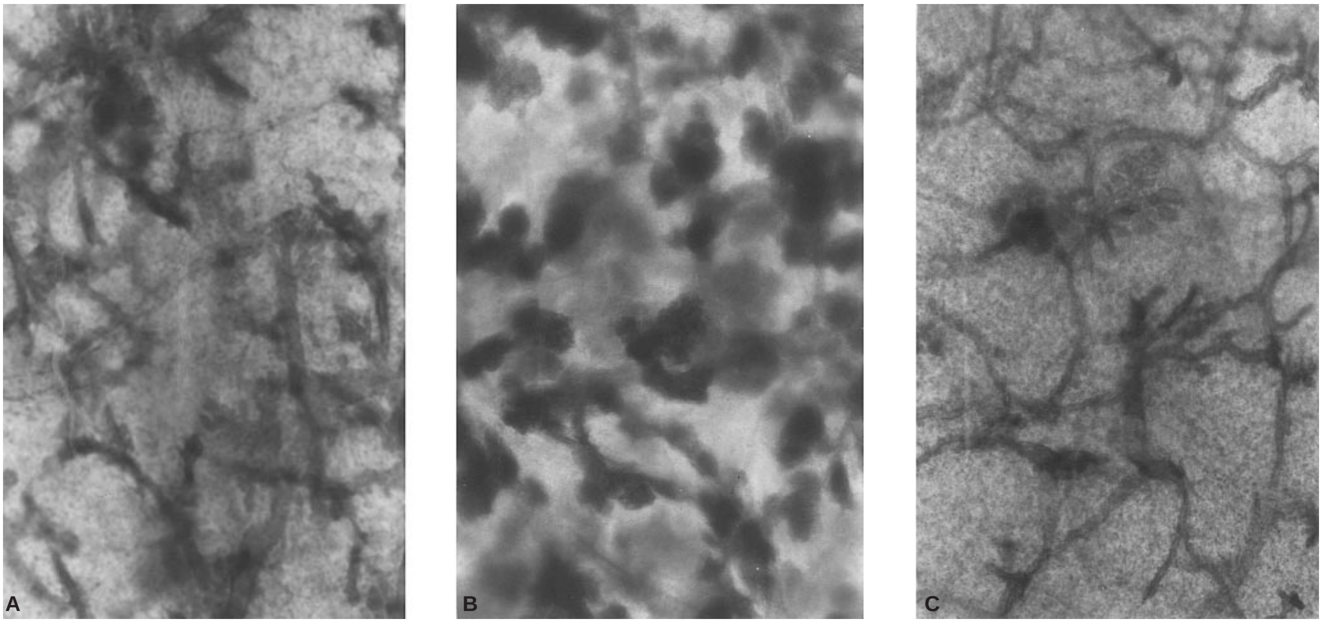

**Figure 2** Mammary wholemount preparations (carmine staining) of the 9th abdominal glands obtained from 6-month-old female rats exposed to vehicle (**A**) 20 µg genistein (**B**) or 20 µg zearalenone (**C**) during a prepubertal period, and to DMBA at the age of 45 days. This figure is representative of 4–5 specimens per group. The wholemounts of the genistein group contained high levels of 2–3 type lobules, and the whole mounts of zearalenone group indicated ductal atrophy, combined with higher level of lobular structures than seen in the vehicle group (but clearly less than in the genistein group). Magnification x 6.3

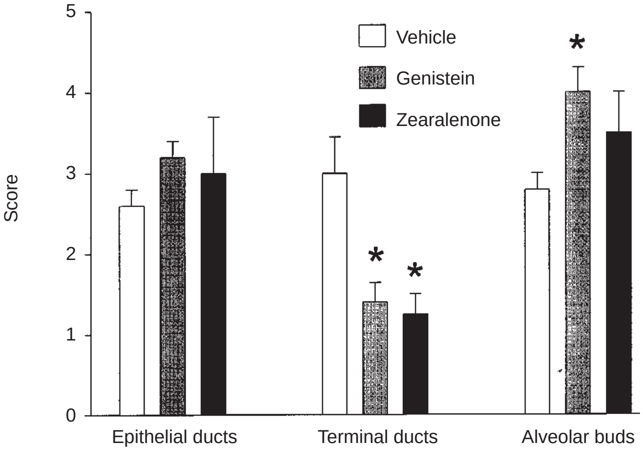

**Figure 3** Density of epithelial ducts, terminal ducts (TDs) and differentiated alveolar buds (ABs) of the 9th abdominal gland obtained from 6-month-old female rats exposed to 20 µg genistein, 20 µg zearalenone, or vehicle during a prepubertal period, and to DMBA at the age of 45 days ($n$ = 4–5 female rats per group). These parameters were evaluated using a scoring system with scores ranging from 0 (absent) to 5 (high), as described in Methods. Means ± s.e.m. are shown. Significantly different from the vehicle group: *$P < 0.05$

genistein-treated group, 40% of the tumours were adenocarcinomas and 60% were non-malignant.

### Oestrogen receptor

The concentrations of ER protein were determined in the mammary glands in rats that were exposed to DMBA 18 weeks prior to sacrifice. The ER levels were similar in the animals treated with genistein (3.4 ± 1.4 fmol $mg^{-1}$ protein) or zearalenone (4.3 ± 2.8 fmol $mg^{-1}$ protein) during the prepubertal period, and did not differ from that of the controls (3.9 ± 1.7 fmol $mg^{-1}$ protein). The total ER levels in the present study were lower than we have seen in mice (Hilakivi-Clarke et al, 1998), but comparable to those seen in rats exposed to carcinogens (Thordarson et al, 1995).

We also determined the percentage of mammary glands that had ER levels higher than 5 fmol $mg^{-1}$. Breast tumours containing ER levels ≥ 5 fmol $mg^{-1}$ protein are often considered ER-positive (Clark and McGuire, 1988; Winstanley et al, 1991). Using this cut-off point the data indicate that the proportion of ER-positive glands was significantly higher in the zearalenone-treated group (43%) than in the genistein- (14%) or vehicle-treated (28%) groups ($\chi^2$ = 17.80, df = 2, $P < 0.01$).

### Mammary whole mounts

Analysis of mammary whole mounts obtained from animals that had been exposed to DMBA 18 weeks prior to sacrifice, indicated that prepubertal exposure to genistein and zearalenone induced distinct differences, when compared with the vehicle-controls (Figure 2). The mammary glands of genistein-treated animals showed the most lobular differentiation. These lobules were of type 2 and 3, indicating a high level of differentiation. Zearalenone-treated glands also showed differentiated lobular structures, although less than that seen in the genistein-treated glands.

According to the scale-based quantitative evaluation, total mammary epithelial density did not differ among the groups (Figure 3). However, the density of terminal ducts was significantly lower in the rats exposed to zearalenone ($P < 0.05$) or genistein ($P < 0.05$) during the prepubertal period than in the vehicle controls ($F(2,11) = 8.30$, $P < 0.006$). The density of alveolar buds, in turn, was significantly higher in the genistein-treated group ($P < 0.05$) than in the controls ($F(2,11) = 3.44$, $P < 0.07$).

*© 1999 Cancer Research Campaign*

### DISCUSSION

Despite the general perception that consumption of soy-based food products protects from breast cancer, the supporting epidemiological evidence is inconsistent. Three studies suggest that soy intake is associated with lower breast cancer risk. A study in Singaporean women found that high soy intake is associated with a lower breast cancer risk among premenopausal, but not postmenopausal, women (Lee et al, 1991). A study in Asian-American women living in Los Angeles or Hawaii indicated that breast cancer risk decreases with increasing frequency of intake of tofu (bean curd) both in pre- and postmenopausal women (Wu et al, 1996). Finally, a study that measured urinary excretion levels of phyto-oestrogens reported that a high excretion of isoflavones (genistein was not included) was associated with a substantial reduction in breast cancer risk (Ingram et al, 1997). A similar, but more recent, study did not find significant differences in urinary excretion levels of daidzein or genistein between breast cancer cases and their controls in Shanghai; however, total isoflavonoid levels were lower in the cases (Zheng et al, 1999). Soy protein intake was similar in these Shanghai women who were newly diagnosed with breast cancer and randomly selected controls. Four other studies also suggest that the risk of breast cancer is not associated with soy consumption (Hirohata et al, 1985; Yuan et al, 1995; Witte et al, 1997). These significant inconsistencies may reflect differences in the end points used for genistein intake (consumption of tofu, miso, or serum isoflavone concentrations). It also is possible that timing of genistein exposure might be critical.

Treatment during prepuberty, with a pharmacological dose of genistein, has been suggested to reduce the subsequent risk to develop breast cancer. Murrill et al (1996) found that a subcutaneous exposure of prepubertal rats on postnatal days 16, 18 and 20 to 500 mg $kg^{-1}$ genistein significantly reduces mammary tumour incidence and multiplicity. However, this dose is 500–5000 times higher than human genistein intake, and may not be directly relevant to human populations. We have used a genistein dose of ~1 mg $kg^{-1}$ body weight, which should approximate the daily genistein consumption in Asia on a mg $kg^{-1}$ body weight basis. If this dose is further adjusted for interspecies surface area differences (Freireich et al, 1966; Clarke, 1997), a human genistein exposure equivalent to 0.143 mg $kg^{-1}$ body weight is obtained, comparable to the exposure to genistein alone in Oriental populations (~ 0.1 mg $kg^{-1}$ body weight). Our data show that when the rats were exposed to this substantially lower genistein dose between postnatal days 7 and 20, they exhibited reduced mammary tumour multiplicity, but no significant change in tumour incidence. Additionally, a significant proportion of the tumours in the genistein group did not proliferate, and 60% of them were not malignant. Thus, prepubertal exposure to genistein not only reduced the subsequent mammary tumour multiplicity, it also reduced the likelihood that a tumour was malignant.

The lack of significance in the tumour incidence and latency in the genistein-exposed rats in the present study, when compared with the previous study (Murrill et al, 1996), is likely to be caused by the use of a low versus high dose of genistein. Genistein displays a convex dose–response curve for oestrogenic activity. Low genistein doses stimulate ER, while higher doses inhibit this receptor's activity (Wang et al, 1996). Inhibition of ER by high genistein doses may be due to inhibition of the tyrosine kinase activity of the epidermal growth factor receptor (EGFR) (Akiyama et al, 1987), which could lead to a reduced phosphorylation of ER. Another means by which pharmacological doses of genistein may affect tumorigenesis is by influencing the reproductive system. The 500 mg $kg^{-1}$ genistein administration to prepubertal rats caused a permanent impairment of the hypothalamic–gonadal axis (Murrill et al, 1996). It also has been reported that 1 mg genistein (~ 100 mg $kg^{-1}$) given to rat pups daily between postnatal days 1 and 10, significantly decreases pituitary responsiveness to gonadotrophin releasing hormone (GnRH) (Faber and Hughes, 1991). A ten times lower genistein dose (100 μg ≈ 10 mg $kg^{-1}$) has the opposite effect, increasing GnRH-induced secretion of luteinizing hormone (Faber and Hughes, 1991). Thus, changes in the magnitude of a genistein exposure may have opposing effects on the reproductive system. We did not perform a detailed analysis of the reproductive system functions, and therefore cannot exclude the possibility that a low genistein dose (1 mg $kg^{-1}$) disrupts reproductive functions. However, we did not observe any differences in the timing of the onset of sexual maturation with prepubertal exposure to either genistein or zearalenone (not shown), which would be one measure of altered reproductive function.

Zearalenone, when administered during the prepubertal period, effectively reduced DMBA-induced mammary tumorigenesis in female rats. Rats that received 20 μg (~ 1 mg $kg^{-1}$) zearalenone every 3–4 days between days 7 and 20, subsequently exhibited a longer latency to tumour appearance, and had a lower tumour incidence than the vehicle-treated controls. In addition, one-third of the tumours did not proliferate. However, histopathological evaluation indicated that all the assessed tumours in the zearalenone group were malignant. These findings contrast with earlier data obtained in rats showing that treatment on postnatal days 7 and 14 with 10 mg $kg^{-1}$ zearalenone increases the incidence of spontaneous mammary tumours (Schoental, 1985). The opposite results may be caused by the tenfold difference in the dose of zearalenone used in the two studies. We also treated the rats with a carcinogen and measured tumorigenesis within the following 18 weeks, while in the other study spontaneously arising tumours appeared when the animals were 2 years of age. These differences also may indicate that prepubertal zearalenone exposure inhibits premenopausal breast cancer (that is mimicked by DMBA), and stimulates the growth of postmenopausal breast cancer (that is mimicked by spontaneous tumours in older rats).

Both genistein and zearalenone act as relatively weak oestrogens. They stimulate the growth of human breast cancer cells in vitro (Martin et al, 1978; Wang et al, 1996), and have similar properties to oestradiol in rodent and human breast tissues (Petrakis et al, 1996; Harding et al, 1997; Santell et al, 1997; Hsieh et al, 1998; McMichael-Phillips et al, 1998). Since oestrogens are thought to increase breast cancer risk (Clarke et al, 1992), it is surprising that genistein or zearalenone in the present and previous study (Murrill et al, 1996) reduced the incidence/multiplicity of mammary tumours. Perhaps oestrogens have opposing effects on breast cancer risk depending on the timing of exposure. In utero exposure to oestrogens, or an exposure during the first week after birth, increases the subsequent incidence of mammary tumours in mice and rats (Bern et al, 1985; Lopez et al, 1988; Hilakivi-Clarke et al, 1997*b*), and possibly also in humans (Ekbom et al, 1992; Michels et al, 1996; Sanderson et al, 1996). However, prepubertal or pubertal treatment with oestradiol reduces mammary tumorigenesis in rats (Nagasawa et al, 1974; Grubbs et al, 1985). An exposure to oestrogens during adulthood increases breast cancer risk in animals (Clarke et al, 1992; Russo et al, 1994), and perhaps in humans (Grodstein et al, 1997). These differences may reflect

 © 1999 Cancer Research Campaign changes in the responsiveness of the mammary gland to ER oestrogens over time.

In the present study, phyto-oestrogen exposure occurred during a prepubertal period when the mammary epithelial cells are thought not to respond fully to oestrogens, e.g. oestrogens do not cause epithelial cell proliferation or alter ER levels (Haslam, 1989). We did not find any evidence that the prepubertal exposure to genistein or zearalenone permanently affected the levels of ER in the mammary gland. However, the mammary glands of the zearalenone-treated rats were significantly more often ER-positive (had ER levels ≥ 5 fmol $mg^{-1}$ protein) than those of the genistein group. Our findings are consistent with the fact that ligands can alter the number/function of a receptor when they are present, and generally do not cause any persistent changes in receptor expression. One exception is an exposure that occurs during fetal life or immediately after birth, which can produce a permanent change (Verhoeven et al, 1982; Bern et al, 1985; Hilakivi-Clarke et al, 1998). However, since a prepubertal treatment with both a low (interacts with ER) and high (interacts with ER and other targets) dose of genistein or zearalenone reduces subsequent mammary tumorigenesis, prepubertal ERs may be involved as mediators of these effects.

Whether or not the effects we report are a direct consequence of changes in oestrogen-regulated gene function, it seems highly likely that the mechanism for prepubertal oestrogen exposure in affecting breast cancer risk involves changes in the mammary epithelial network. We have proposed that the increased number of terminal end buds and their reduced differentiation to alveolar buds, plays a critical role in increasing breast cancer risk following a perinatal oestrogen exposure (Hilakivi-Clarke et al, 1997*a*). Terminal end buds, and to a lesser degree terminal buds, are the primary sites of carcinogen action in the mammary gland (Russo and Russo, 1987). Consistent with this hypothesis, prepubertal exposure to a pharmacological genistein dose increases differentiation of terminal end buds at the time when DMBA is administered (Murrill et al, 1996). In our study, a more physiological dose of genistein also increased differentiated alveolar buds, when determined 18 weeks after DMBA exposure.

In summary, prepubertal exposure to a low dose of genistein or zearalenone reduces the risk to develop mammary tumours in rats. The mediating mechanisms remain to be established, but are likely to include changes in the differentiation of mammary epithelial tree and may reflect events mediated through activation of the ER.

### REFERENCES

Akiyama T, Ishida J, Nakagawa S, Ogawa H, Watanabe S, Itou N, Shibata M and Fukami Y (1987) Genistein, a specific inhibitor of tyrosine-specific protein kinase. *J Biol Chem* **262**: 5592–5595

Barnes S (1997) The chemopreventive properties of soy isoflavonoids in animal models of breast cancer. *Breast Cancer Res Treat* **46**: 169–179

Bern HA, Mills KT and Edery M (1985) Estrogen-associated defects in rodent mammary gland development. In: *Estrogens in the Environment*, McLachlan JA (ed), pp. 319–326 Elsevier: Amsterdam

Burgess LW, Nelson PE and Toussoun TA (1982) Characterization, geographic distribution and ecology of Fusarium crookwellense sp. nov. *Trans Br Mycol Soc* **79**: 497–505

Clark GM and McGuire WL (1988) Steroid receptors and other prognostic factors in primary breast cancer. *Semin Oncol* **15**: 20–25

Clarke R (1997) Issues in experimental design and analysis in the study of experimental cytotoxic agents in vivo in breast cancer and other models. *Breast Cancer Res Treat* **46**: 255–278

Clarke R, Dickson RB and Lippman ME (1992) Hormonal aspects of breast cancer. Growth factors, drugs and stromal interactions. *Crit Rev Oncol Hematol* **12**: 1–23

Collins BM, McLachlan JA and Arnold SF (1997) The estrogenic and antiestrogeneic activities of phytochemicals with the human estrogen receptor expressed in yeast. *Steroids* **62**: 365–372

Ekbom A, Trichopoulos D, Adami HO, Hsieh CC and Lan SJ (1992) Evidence of prenatal influences on breast cancer risk. *Lancet* **340**: 1015–1018

Faber KA and Hughes CLJ (1991) The effect of neonatal exposure to diethylstilbestrol, genistein, and zearalenone on pituitary responsiveness and sexually dimorphic nucleus volume in the castrated adult rat. *Biol Reprod* **45**: 649–653

Freireich EJ, Gehan EA, Rall DP, Schmidt LH and Skipper HE (1966) Quantitative comparison of toxicity of anticancer agents in the mouse, rat, hamster, dog, monkey and man. *Cancer Chemother Rep* **50**: 219–244

Fukutake M, Takahashi M, Ishida K, Kawamura H, Sugimura T and Wakabayashi K (1996) Quantification of genistein and genistin in soybeans and soybean products. *Food Chem Toxicol* **34**: 457–461

Gotoh T, Yamada K, Yin H, Ito A, Kataoka T and Dohi K (1998) Chemoprevention of N-nitroso-N-methylurea-induced rat mammary carcinogenesis by soy foods or biochanin A. *Jpn J Cancer Res* **89**: 137–142

Grodstein F, Stampfer MJ, Colditz GA, Willett WC, Manson JE, Joffe M, Rosner B, Fuchs C, Hankinson SE, Hunter DJ, Hennekens CH and Speizer FE (1997) Postmenopausal hormone therapy and mortality. *N Eng J Med* **336**: 1769–1775

Grubbs CJ, Farneli DR, Hill DL and McDonough KC (1985) Chemoprevention of n-nitro-n-methylurea-induced mammary cancers by pretreatment with 17beta-estradiol and progesterone. *J Natl Cancer Inst* **74**: 927–931

Hagler WM, Tyczkowska K and Hamilton PB (1984) Simultaneous occurrence of deoxynivalenol, zearalenone, and aflatoxin in 1982 scabby wheat from the Midwestern United States. *Appl Environ Microbiol* **47**: 151–154

Hanfelt JJ (1997) Statistical approaches to experimental design and data analysis of in vivo studies. *Breast Cancer Res Treat* **46**: 279–302

Harding C, Tetlow L, McMichael Phillips D, Osundeko O, Potten CS and Bundred NJ (1997) Oestrogenic effects of soy on nipple aspirate fluid. *Breast Cancer Res Treat* **46**: 80-Abstract

Haslam SZ (1988) Progesterone effects on deoxyribonucleic acid synthesis in normal mouse mammary glands. *Endocrinology* **122**: 464–470

Haslam S (1989) The ontogeny of mouse mammary gland responsiveness to ovarian steroid hormones. *Endocrinology* **125**: 2766–2772

Hawrylewicz EJ, Huang HH and Blair WH (1991) Dietary soybean isolate and methionine supplementation affect mammary tumor progression in rats. *J Nutr* **121**: 1693–1698

Hilakivi-Clarke L, Cho E, Raygada M and Kenney N (1997*a*) Alterations in mammary gland development following neonatal exposure to estradiol, transforming growth factor alpha, and estrogen receptor antagonist ICI 182, 780. *J Cell Physiol* **170**: 279–289

Hilakivi-Clarke L, Clarke R, Onojafe I, Raygada M, Cho E and Lippman ME (1997*b*) A maternal diet high in n-6 polyunsaturated fats alters mammary gland development, puberty onset, and breast cancer risk among female rat offspring. *Proc Natl Acad Sci USA* **94**: 9372–9377

Hilakivi-Clarke LA, Raygada M, Stoica A and Martin M-B (1998) Consumption of a high-fat diet during pregnancy alters estrogen receptor content, protein kinase C activity and morphology of mammary gland in the mother and her female offspring. *Cancer Res* **58**: 654–660

Hirohata T, Shigematsu T, Nomura AMY, Nomura Y, Horie A and Hirohata I (1985) Occurrence of breast cancer in relation to diet and reproductive history: a case-control study in Fukuoka, Japan. *Natl Cancer Inst Monogr* **69**: 187–190

Hsieh CY, Santell RC, Haslam SZ and Helferich WG (1998) Estrogenic effects of genistein on the growth of estrogen receptor-positive human breast cancer (MCF-7) cells in vitro and in vivo. *Cancer Res* **58**: 3833–3838

Ingram D, Sanders K, Kolybaba M and Lopez D (1997) Case-control study of phytoestrogens and breast cancer. *Lancet* **350**: 990–994

Kuiper-Goodman T (1990) Uncertainties in the risk assessment of three mycotoxins: aflatoxin, ochratoxin, and zearalenone. *Can J Physiol Pharm* **68**: 1017–1024

Lee HP, Gourley L, Duffy SW, Esteve J, Lee J and Day NE (1991) Dietary effects on breast cancer risk in Singapore. *Lancet* **337**: 1197–1200

Lopez J, Ogren L, Verjan R and Talamantes F (1988) Effects of perinatal exposure to a synthetic estrogen and progestin on mammary tumorigenesis in mice. *Teratology* **38**: 129–134

Lu LJ, Broemeling L, Marshall M and Ramanujam VM (1995) A simplified method to quantify isoflavones in commercial soybean diets and human urine after legume consumption. *Cancer Epidemiol Biomarkers Prev* **4**: 497–503

*© 1999 Cancer Research Campaign*

Luo Y, Yoshizawa T and Katayama T (1990) Comparative study on the natural occurrence of fusarium mycotoxins (trichothecenes and zearalenone) in corn and wheat from high- and low-risk areas for human esophageal cancer in China. *Appl Environ Microbiol* **56**: 3723–3726
McMichael-Phillips DF, Harding C, Morton M, Roberts SA, Howell A, Potten CS and Bundred NJ (1998) Effect of soy-protein supplementation on epithelial proliferation in the histologically normal human breast. *Am J Clin Nutr* **68** (suppl), 1431S–1436S
Martin PM, Horwitz KB, Ryan DS and McGuire W (1978) Phytoestrogen interaction with estrogen receptors in human breast cancer cells. *Endocrinology* **103**: 1860–1867
Messina M, Persky V, Setchell KDR and Barnes S (1994) Soy intake and breast cancer: a review of the in vitro and in vivo data. *Nutr Cancer* **21**: 113–131
Michels KB, Trichopoulos D, Robins JM, Rosner BA, Manson JE, Hunter D, Colditz GA, Hankinson SE, Speizer FE and Willett WC (1996) Birth weight as a risk factor for breast cancer. *Lancet* **348**: 1542–1546
Murrill WB, Brown NM, Zhang JX, Manzolillo PA, Barnes S and Lamartiniere CA (1996) Prepubertal genistein exposure suppresses mammary cancer and enhances gland differentiation in rats. *Carcinogenesis* **17**: 1451–1457
Nagasawa H, Yanai R, Shonodo M, Nakamura T and Tanabe Y (1974) Effect of neonatally administered estrogen and prolactin on normal and neoplastic mammary growth and serum estradiol-17 level in rats. *Cancer Res* **34**: 2643–2646
Nelson J, Clarke R, Dickson GR, Van Den Berg HW and Murphy RF (1986) The effects of Mg2+ ions or EDTA on nuclear integrity and apparent subcellular distribution of unoccupied oestrogen receptors in breast cancer cells. *J Steroid Biochem* **25**: 619–626
Nomura A, Henderson BE and Lee J (1978) Breast cancer and diet among the Japanese in Hawaii. *Am J Clin Nutr* **31**: 2020–2025
Petrakis NL, Barnes S, King EB, Lowenstein J, Wiencke J, Lee MM, Miike R, Kirk M and Coward L (1996) Stimulatory influence of soy protein isolate on breast secretion in pre- and postmenopausal women. *Cancer Epidemiol Biomark Prev* **5**: 785–794
Ralston AT (1978) Effect of zearalanol on weaning weight of male calves. *J Anim Sci* **47**: 1203–1206
Russo IH, Medado J and Russo J (1994) Endocrine influences on the mammary gland. In *Integument and Mammary Glands*. Eds TC Jones, U Mohr & RD Hunt. Berlin: Springer-Verlag. pp. 252–266
Russo J and Russo IH (1987) Biological and molecular bases of mammary carcinogenesis. *Lab Invest* **57**: 112–137
Sanderson M, Williams M, Malone KE, Stanford JL, Emanuel I, White E and Daling JR (1996) Perinatal factors and risk of breast cancer. *Epidemiology* **7**: 34–37
Santell RC, Chang YC, Nair MG and Helferich WG (1997) Dietary genistein exerts estrogeneic effects upon the uterus, mammary gland and the hypothalamic/pituitary axis in rats. *J Nutr* **127**: 263–269
Schoental R (1974) Role of podophyllotoxin in the bedding and dietary zearalenone on incidence of ‘spontaneous’ tumors in laboratory animals. *Cancer Res* **34**: 2419
Schoental R (1985) Trichothecenes, Zearalenone, and other carcinogenic metabolites of fusarium and related microfungi. *Adv Cancer Res* **45**: 217–290
Seow A, Shi C-Y, Franke A, Hankin JH, Lee HP and Yu MC (1998) Isoflavoid levels in spot urine are associated with frequency of dietary soy intake in a population-based sample of middle-aged and older Chinese in Singapore. *Cancer Epidemiol Biomarkers Prev* **7**: 135–140
Setchell KDR, Borriello SP, Hulme P, Kirk DN and Axelson M (1984) Nonsteroidal estrogens of dietary origin: possible roles in hormone-dependent disease. *Am J Clin Nutr* **40**: 569–578
Snedecor GW (1988) *Statistical Methods*, 8th Edn. Iowa State University Press: Ames, Iowa
Thordarson G, Jin E, Guzman RC, Swanson SM, Nandi S and Talamantes F (1995) Refractoriness to mammary tumorigenesis in parous rats: is it caused by persistent changes in the hormonal environment or permanent biochemical alterations in the mammary epithelia? *Carcinogenesis* **16**: 2847–2853
Verhoeven G, Vandoren G, Heyns W, Kuhn ER, Janssens JP, Teuwen D, Goddeeris E, Lesaffre E and DeMoor P (1982) Incidence, growth and estradiol-receptor levels of 7,12-dimethylbenz(alpha)antracene-induced mammary tumours in rats: effects of neonatal sex steroids and oestradiol implants. *J Endocrinol* **95**: 357–368
Wang TT, Sathyamoorthy N and Phang JM (1996) Molecular effects of genistein on estrogen receptor mediated pathways. *Carcinogenesis* **17**: 271–275
Wiggins JP, Rothenbacher H, Wilson LL, Martin RJ, Wangness PJ and Ziegler JH (1979) Growth and endocrine responses of lambs to zearanol implants: effects of preimplant growth rate and breed of sire. *J Anim Sci* **49**: 291–297
Winstanley J, Cooke T, George WD, Murray G, Holt S, Croton R, Griffiths K and Nicholson R (1991) The long term prognostic significance of oestrogen receptor analysis in early carcinoma of the breast. *Br J Cancer* **64**: 99–101
Witte JS, Ursin G, Siemiatycki J, Thompson WD, Paganini-Hill A and Haile RW (1997) Diet and premenopausal bilateral breast cancer: A case-control study. *Breast Cancer Res Treat* **42**: 243–251
Wu AH, Ziegler RG, Horn-Ross PL, Nomura AMY, West DW, Kolonel LN, Rosenthal JF, Hoover RN and Pike MC (1996) Tofu and risk of breast cancer in Asian-Americans. *Cancer Epidemiol Biomarkers Prev* **5**: 901–906
Wu AH, Ziegler RG, Nomura AMY, West DW, Kolonel LN, Horn-Ross PL, Hoover RN and Pike MC (1998) Soy intake and risk of breast cancer in Asians and Asian-Americans. *Am J Clin Nutr* **68** (Suppl), 1437S–1443S
Yuan JM, Wang QS, Ross RK, Henderson BE and Yu MC (1995) Diet and breast cancer in Shanghai and Tianjin, China. *Br J Cancer* **71**: 1353–1358
Zava DT and Duwe G (1997) Estrogenic and antiproliferative properties of genistein and other flavonoids in human breast cancer cells in vitro. *Nutr Cancer* **27**: 31–40
Zheng W, Dai Q, Cluster LJ, Shu XO, Wen WQ, Jin F and Franke AA (1999) Urinary excretion of isoflavonoids and the risk of breast cancer. *Cancer Epidemiol Biomarkers Prev* **8**: 35–40

*© 1999 Cancer Research Campaign*
